# Monitoring State-Level Changes in Walking, Biking, and Taking Public Transit to Work — American Community Survey, 2006 and 2017

**DOI:** 10.5888/pcd17.200097

**Published:** 2020-10-01

**Authors:** Geoffrey P. Whitfield, Brian McKenzie, Kaitlin A. Graff, Susan A. Carlson

**Affiliations:** 1 Physical Activity and Health Branch, Division of Nutrition, Physical Activity, and Obesity, National Center for Chronic Disease Prevention and Health Promotion, Centers for Disease Control and Prevention, Atlanta, Georgia; 2 Journey to Work and Migration Statistics Branch, US Census Bureau, Washington, District of Columbia

## Abstract

**Introduction:**

Active commuting to work is one way people can be physically active and can be influenced by state-level initiatives. The American Community Survey (ACS) is a potential data source to evaluate changes in active commuting at the state level, but state-level changes have not been well documented. We examined state-level changes in estimates of walking, biking, and taking transit to work (combined and separately) among employed persons between 2006 and 2017.

**Methods:**

Data were from the ACS, a nationally representative annual household survey from the US Census Bureau. We estimated state-level prevalence of walking, bicycling, or taking transit to work (separately and in combination) in 2006 and 2017 and tested differences by year.

**Results:**

The prevalence of active commuting to work varied widely among states (2017 range: 1.7% in Alabama and Mississippi to 35.0% in New York). Changes from 2006 to 2017 also varied, with 8 states exhibiting a significant increase (Massachusetts [2.7 percentage points], New York [2.2], Hawaii [1.6], Illinois [1.3], Washington [1.3], New Jersey [1.2], Virginia [0.9], and Michigan [0.4]), and 12 exhibiting a significant decrease (South Dakota [−1.9], Idaho [−1.3], New Hampshire [−1.3], Wisconsin [−1.1], Maryland [−1.0], Nevada [−0.9], Ohio [−0.8], Mississippi [−0.6], Texas [−0.6], Florida [−0.5], Georgia [−0.4], and Indiana [−0.4]). The contributions of walking, bicycling, and taking transit also varied by state.

**Conclusion:**

Active commuting remains relatively rare across states. States pursuing initiatives to support active transportation may consider using ACS to monitor and evaluate changes in active commuting.

**Summary**

**What is already known on this topic?**

Active commuting to work is one way people can be physically active and is influenced by state-level initiatives. Active commuting by walking, bicycling, or using public transit is rare in the United States and varies by state.

**What is added by this report?**

Active commuting to work (combined and individual modes) remained rare in most states; changes in active commuting have been inconsistent across states. Many significant changes were of small magnitude.

**What are the implications for public health practice?**

The American Community Survey is useful for monitoring and evaluating state-level active commuting to work. When using ACS, careful consideration of included constructs, change measures, time period, and geographic levels is needed.

## Introduction

Physical activity participation confers many health benefits, including short-term improvements such as reduced anxiety and improved sleep and longer-term improvements such as reduced risk for cardiovascular disease, diabetes, and several cancers (1). To attain substantial health benefits, the *Physical Activity Guidelines for Americans*, second edition, recommends adults do at least 150 minutes a week of moderate-intensity equivalent aerobic activity (1). Active commuting by walking, bicycling, or using public transit (eg, walking to and from transit stops [2]) can help adults meet physical activity guidelines and is a component of national physical activity guidance documents such as the *National Physical Activity Plan* and *Step it Up! The Surgeon General’s Call to Action to Promote Walking and Walkable Communities* (3,4). Active commuting is also a component of the Active People, Healthy Nation initiative of the Centers for Disease Control and Prevention (CDC), which aims to get 27 million Americans more active by 2027 (5).

State-level policies, programs, and practices can influence active commuting. For example, many states have adopted Complete Streets policies that help provide safe roadway access for all users at all ability levels, including pedestrians, bicyclists, and those riding public transportation (6,7). Additionally, states have administered Safe Routes to School programs (8,9) and may have policies that support transit-oriented development (10,11). State-level estimates of active commuting over time are therefore important for supporting decision making and evaluating progress; however, few nationally consistent data sources to monitor and evaluate state-level active commuting exist.

The American Community Survey (ACS) (12) provides annual, publicly available information on active commuting to work. With a large, nationally representative sample, high response rate, and consistent annual measurement spanning 12 years, ACS is a possible data source for state-level monitoring of active commuting to work. Several reports have examined state-level changes in active commuting in ACS, but have omitted 1 or more active modes or lacked statistical testing (13,14). The purpose of this article is to explore the usefulness of ACS for monitoring state-level changes in active commuting to work. To do this, we will examine differences in estimates of walking, biking, and taking public transit to work (combined and separately) by region and state in 2006 and 2017, the longest interval in ACS that uses consistent sampling.

## Methods

ACS is a nationally representative, continuous survey conducted by the US Census Bureau since 2005. These analyses use 2006 as baseline because of inclusion of group quarters (eg, college dormitories) in ACS after 2005. The ACS sampling frame is developed from the Census Master Address File, from which is drawn a stratified sample of housing units and group quarters in every county or county-equivalent in the United States. Information is collected on all residents in a sampled housing unit, or single residents of a sampled group quarters location (12). Individual-level data for 2006 and 2017 were obtained from the Census Bureau’s ACS website. These years were chosen to allow the longest time interval between measurements while using a consistent sampling strategy. In 2006, approximately 2.0 million household and group quarters interviews yielded data for approximately 3.0 million people; in 2017, 2.1 million interviews yielded data on 3.2 million people (15). Response rates were 97.5% in 2006 and 93.7% in 2017.

For each employed participant aged 16 years or older, the ACS questionnaire asked, “How did this person usually get to work last week? If this person usually used more than one method of transportation during the trip, mark the box of the one used for most of the distance” (16). Participants then chose from a list of common commute modes, including working from home. Participants who reported walking, bicycling, or using public transit (including “bus or trolley bus, streetcar or trolley car, subway or elevated, railroad, and ferryboat”) (16) as their single, primary mode were classified as active commuters. Public transit was classified as an active mode because transit riders tend to be physically active while getting to and from transit stops (1,2).

The prevalence of combined active commuting to work and that of each of the 3 active modes was estimated by state of residence and year. Washington, District of Columbia (DC), was included in this analysis; however, because Washington, DC, functions more as a city than a state, comparisons between DC and states should be made with caution. For comparison purposes, we estimated the prevalence of combined active commuting and each active mode for the United States and the 4 Census regions (Northeast, Midwest, South, and West), as well as the median state-level prevalence of each measure for the nation and the 4 Census regions.

Differences between 2006 and 2017 were tested with adjusted Wald tests and deemed significant if *P* was less than .05. All analyses were performed in Stata 13 (StataCorp LLC) and used population weights and successive difference replication for variance estimation based on ACS analytic guidelines (17). To replicate analyses that might be performed by state-level professionals, who would be interested primarily in evaluating changes for 1 state, we did not adjust *P* values for multiple comparisons. Results described here were significant unless otherwise noted.

## Results

In 2017, the prevalence of active commuting to work varied widely across states, from 1.7% in both Alabama and Mississippi to 35.0% in New York (Table 1). The regional prevalence varied from 4.2% in the South to 19.6% in the Northeast, and the national prevalence was 8.2%. When examining each mode of active commuting separately, the prevalence of walking to work varied from 1.3% in Alabama to 6.9% in Alaska. The regional prevalence varied from 1.8% in the South to 4.4% in the Northeast, and the national prevalence was 2.6%. Bicycling to work varied from less than 0.1% in Mississippi to 2.2% in Oregon. The regional prevalence varied from 0.3% in the South to 0.9% in the West, and the national prevalence was 0.6%. Taking transit to work varied from 0.2% in South Dakota to 28.2% in New York. The regional prevalence varied from 2.0% in the South to 14.5% in the Northeast, and the national prevalence was 5.0%. For comparison purposes, the medians of state-level prevalence estimates for the nation and each Census region are presented in Table 2.

**Table 1:** Prevalence of Active Commuting to Work Among Employed Residents Aged 16 Years or Older, by Census Region and State, American Community Survey, 2006 and 2017

Region/State	Combined^a^, % (SE)		Walk, % (SE)		Bike, % (SE)		Transit, % (SE)	
	2006	2017	2006	2017	2006	2017	2006	2017
**Northeast**	**18.2 (0.1)**	**19.6 (0.1)**	**4.7 (0.1)**	**4.4 (0.1)**	**0.4 (0.0)**	**0.6 (0.0)**	**13.1 (0.1)**	**14.5 (0.1)**
Connecticut	7.2 (0.3)	7.7 (0.2)	3.1 (0.2)	2.9 (0.2)	0.2 (0.0)	0.3 (0.1)	3.9 (0.2)	4.5 (0.2)
Massachusetts	13.4 (0.3)	16.1 (0.2)	4.3 (0.1)	4.8 (0.1)	0.5 (0.1)	0.9 (0.1)	8.6 (0.2)	10.4 (0.2)
Maine	5.6 (0.3)	4.9 (0.4)	4.4 (0.3)	3.8 (0.3)	0.4 (0.1)	0.5 (0.1)	0.7 (0.2)	0.6 (0.1)
New Hampshire	4.4 (0.4)	3.1 (0.3)	3.7 (0.4)	2.2 (0.2)	0.1 (0.1)	0.2 (0.1)	0.6 (0.1)	0.8 (0.1)
New Jersey	14.0 (0.2)	15.2 (0.2)	3.4 (0.1)	3.0 (0.1)	0.4 (0.0)	0.3 (0.0)	10.2 (0.2)	11.9 (0.2)
New York	32.8 (0.2)	35.0 (0.2)	6.3 (0.1)	6.1 (0.1)	0.4 (0.0)	0.7 (0.0)	26.2 (0.2)	28.2 (0.2)
Pennsylvania	9.6 (0.2)	9.6 (0.2)	4.1 (0.1)	3.6 (0.1)	0.3 (0.0)	0.6 (0.1)	5.2 (0.1)	5.4 (0.1)
Rhode Island	5.6 (0.4)	5.9 (0.4)	2.9 (0.3)	3.6 (0.3)	0.2 (0.1)	0.2 (0.1)	2.5 (0.2)	2.1 (0.2)
Vermont	7.3 (0.7)	7.1 (0.6)	5.6 (0.6)	5.1 (0.5)	0.6 (0.2)	0.5 (0.2)	1.1 (0.2)	1.4 (0.4)
**Midwest**	**6.0 (0.1)**	**5.9 (0.1)**	**2.8 (0.0)**	**2.5 (0.0)**	**0.4 (0.0)**	**0.5 (0.0)**	**2.8 (0.0)**	**3.0 (0.0)**
Iowa	5.1 (0.2)	4.5 (0.2)	3.7 (0.2)	3.0 (0.2)	0.5 (0.1)	0.6 (0.1)	0.9 (0.1)	0.9 (0.1)
Illinois	11.8 (0.2)	13.1 (0.2)	2.8 (0.1)	2.9 (0.1)	0.5 (0.0)	0.7 (0.0)	8.5 (0.1)	9.6 (0.2)
Indiana	3.6 (0.1)	3.3 (0.1)	2.3 (0.1)	1.9 (0.1)	0.4 (0.0)	0.4 (0.0)	1.0 (0.1)	0.9 (0.1)
Kansas	3.4 (0.2)	3.2 (0.2)	2.6 (0.2)	2.3 (0.2)	0.3 (0.1)	0.4 (0.1)	0.5 (0.1)	0.5 (0.1)
Michigan	3.7 (0.1)	4.1 (0.1)	2.2 (0.1)	2.3 (0.1)	0.4 (0.0)	0.5 (0.0)	1.1 (0.1)	1.3 (0.1)
Minnesota	6.8 (0.2)	6.8 (0.2)	3.0 (0.1)	2.6 (0.1)	0.7 (0.1)	0.8 (0.1)	3.1 (0.2)	3.4 (0.2)
Missouri	3.6 (0.1)	3.4 (0.2)	2.1 (0.1)	1.8 (0.1)	0.2 (0.0)	0.2 (0.0)	1.4 (0.1)	1.3 (0.1)
Nebraska	4.3 (0.3)	3.8 (0.3)	3.5 (0.3)	2.8 (0.2)	0.4 (0.1)	0.3 (0.1)	0.5 (0.1)	0.7 (0.1)
North Dakota	4.8 (0.5)	5.3 (0.5)	4.0 (0.4)	4.2 (0.4)	0.3 (0.1)	0.7 (0.2)	0.4 (0.2)	0.4 (0.2)
Ohio	4.7 (0.1)	3.9 (0.1)	2.5 (0.1)	2.1 (0.1)	0.2 (0.0)	0.3 (0.0)	1.9 (0.1)	1.5 (0.1)
South Dakota	5.7 (0.6)	3.8 (0.4)	4.5 (0.5)	3.2 (0.3)	0.7 (0.2)	0.4 (0.1)	0.5 (0.2)	0.2 (0.1)
Wisconsin	6.3 (0.2)	5.2 (0.2)	3.6 (0.2)	3.0 (0.1)	0.7 (0.1)	0.6 (0.1)	1.9 (0.1)	1.6 (0.1)
**South**	**4.4 (0.0)**	**4.2 (0.0)**	**1.9 (0.0)**	**1.8 (0.0)**	**0.3 (0.0)**	**0.3 (0.0)**	**2.3 (0.0)**	**2.0 (0.0)**
Alabama	1.6 (0.1)	1.7 (0.1)	1.1 (0.1)	1.3 (0.1)	0.1 (0.0)	0.1 (0.0)	0.4 (0.1)	0.4 (0.1)
Arkansas	2.4 (0.2)	2.2 (0.2)	1.8 (0.2)	1.7 (0.2)	0.2 (0.1)	0.2 (0.1)	0.5 (0.1)	0.3 (0.1)
Delaware	5.2 (0.6)	5.2 (0.4)	2.2 (0.4)	2.3 (0.3)	0.4 (0.2)	0.4 (0.1)	2.7 (0.3)	2.5 (0.3)
Florida	4.2 (0.1)	3.7 (0.1)	1.7 (0.1)	1.4 (0.1)	0.5 (0.0)	0.6 (0.0)	2.0 (0.1)	1.7 (0.1)
Georgia	4.3 (0.1)	3.9 (0.1)	1.8 (0.1)	1.6 (0.1)	0.2 (0.0)	0.3 (0.0)	2.3 (0.1)	2.1 (0.1)
Kentucky	3.3 (0.2)	3.5 (0.2)	2.2 (0.1)	2.2 (0.1)	0.1 (0.0)	0.2 (0.0)	1.0 (0.1)	1.1 (0.1)
Louisiana	3.5 (0.2)	3.3 (0.2)	1.9 (0.1)	1.6 (0.1)	0.4 (0.1)	0.6 (0.1)	1.2 (0.1)	1.1 (0.1)
Maryland	11.8 (0.3)	10.8 (0.2)	2.6 (0.1)	2.0 (0.1)	0.2 (0.0)	0.4 (0.0)	8.9 (0.2)	8.3 (0.2)
Mississippi	2.3 (0.2)	1.7 (0.1)	1.8 (0.2)	1.4 (0.1)	0.1 (0.0)	0.0 (0.0)	0.4 (0.1)	0.3 (0.1)
North Carolina	3.0 (0.1)	2.9 (0.1)	1.7 (0.1)	1.7 (0.1)	0.2 (0.0)	0.2 (0.0)	1.0 (0.1)	1.0 (0.1)
Oklahoma	2.6 (0.1)	2.4 (0.2)	1.9 (0.1)	1.8 (0.2)	0.2 (0.0)	0.3 (0.1)	0.5 (0.1)	0.3 (0.1)
South Carolina	2.6 (0.1)	2.8 (0.1)	1.7 (0.1)	2.1 (0.1)	0.3 (0.0)	0.2 (0.0)	0.6 (0.1)	0.5 (0.1)
Tennessee	2.4 (0.1)	2.1 (0.1)	1.5 (0.1)	1.4 (0.1)	0.1 (0.0)	0.1 (0.0)	0.8 (0.1)	0.6 (0.1)
Texas	3.8 (0.1)	3.2 (0.1)	1.9 (0.0)	1.5 (0.0)	0.2 (0.0)	0.3 (0.0)	1.7 (0.0)	1.4 (0.1)
Virginia	6.5 (0.2)	7.3 (0.2)	2.2 (0.1)	2.7 (0.1)	0.2 (0.0)	0.4 (0.0)	4.1 (0.2)	4.3 (0.1)
Washington, DC	52.3 (1.2)	49.4 (1.0)	11.5 (0.7)	12.5 (0.8)	1.8 (0.3)	5.4 (0.5)	39.0 (1.3)	31.5 (1.0)
West Virginia	4.0 (0.3)	3.9 (0.3)	3.1 (0.3)	2.9 (0.3)	0.1 (0.0)	0.1 (0.0)	0.9 (0.1)	0.9 (0.1)
**West**	**7.9 (0.1)**	**7.9 (0.1)**	**2.9 (0.0)**	**2.7 (0.0)**	**0.8 (0.0)**	**0.9 (0.0)**	**4.1 (0.0)**	**4.2 (0.0)**
Alaska	11.0 (0.7)	10.1 (0.7)	9.0 (0.6)	6.9 (0.5)	1.1 (0.2)	0.8 (0.2)	0.9 (0.2)	2.4 (0.5)
Arizona	5.1 (0.2)	4.6 (0.2)	2.3 (0.1)	1.9 (0.1)	0.7 (0.1)	0.8 (0.1)	2.1 (0.1)	1.9 (0.1)
California	8.5 (0.1)	8.4 (0.1)	2.7 (0.1)	2.5 (0.0)	0.8 (0.0)	0.9 (0.0)	5.0 (0.1)	5.0 (0.1)
Colorado	7.3 (0.2)	7.0 (0.2)	3.1 (0.2)	2.8 (0.1)	1.1 (0.1)	1.0 (0.1)	3.0 (0.2)	3.2 (0.1)
Hawaii	10.7 (0.5)	12.3 (0.6)	4.5 (0.3)	5.2 (0.4)	0.8 (0.2)	1.0 (0.2)	5.4 (0.4)	6.1 (0.4)
Idaho	4.9 (0.3)	3.7 (0.3)	3.5 (0.3)	2.4 (0.2)	0.6 (0.1)	0.8 (0.1)	0.9 (0.1)	0.5 (0.1)
Montana	7.3 (0.5)	6.9 (0.5)	4.9 (0.4)	4.8 (0.4)	1.7 (0.3)	1.1 (0.2)	0.7 (0.1)	1.0 (0.2)
Nevada	6.3 (0.3)	5.4 (0.3)	2.1 (0.2)	1.9 (0.1)	0.6 (0.1)	0.4 (0.1)	3.6 (0.2)	3.1 (0.2)
New Mexico	3.8 (0.3)	3.8 (0.3)	2.3 (0.2)	2.2 (0.2)	0.5 (0.1)	0.6 (0.1)	1.0 (0.1)	1.0 (0.1)
Oregon	10.0 (0.3)	10.3 (0.3)	3.8 (0.2)	3.8 (0.2)	1.7 (0.1)	2.2 (0.1)	4.6 (0.2)	4.3 (0.2)
Utah	5.9 (0.3)	5.5 (0.3)	2.8 (0.2)	2.3 (0.2)	0.6 (0.1)	0.7 (0.1)	2.4 (0.2)	2.5 (0.2)
Washington	9.3 (0.2)	10.5 (0.2)	3.3 (0.1)	3.3 (0.1)	0.7 (0.1)	0.7 (0.1)	5.2 (0.2)	6.5 (0.2)
Wyoming	5.7 (0.6)	5.6 (0.7)	3.2 (0.5)	4.2 (0.6)	1.4 (0.3)	0.9 (0.2)	1.1 (0.2)	0.6 (0.2)
**Nation**	**8.1 (0.0)**	**8.2 (0.0)**	**2.9 (0.0)**	**2.6 (0.0)**	**0.4 (0.0)**	**0.6 (0.0)**	**4.8 (0.0)**	**5.0 (0.0)**

Abbreviation: SE, standard error.

a Combined active commuting is the sum of commuting by walking, bicycling, or public transit.

**Table 2:** Median State-Level Prevalence of Active Commuting to Work for the United States and by Census Region, American Community Survey, 2006 and 2017

Area	Combined^a^		Walk		Bike		Transit	
	2006, %	2017, %	2006, %	2017, %	2006, %	2017, %	2006, %	2017, %
**National**	5.2	4.9	2.8	2.5	0.4	0.5	1.4	1.4
**Census region**								
Northeast	7.3	7.7	4.1	3.6	0.4	0.5	3.9	4.5
Midwest	4.8	4.0	2.9	2.7	0.4	0.5	1.1	1.1
South	3.7	3.4	1.9	1.8	0.2	0.3	1.0	1.1
West	6.8	6.3	3.2	2.7	0.8	0.9	2.7	2.8

a Combined active commuting is the sum of commuting by walking, bicycling, and taking public transit.

**Figure 1:**
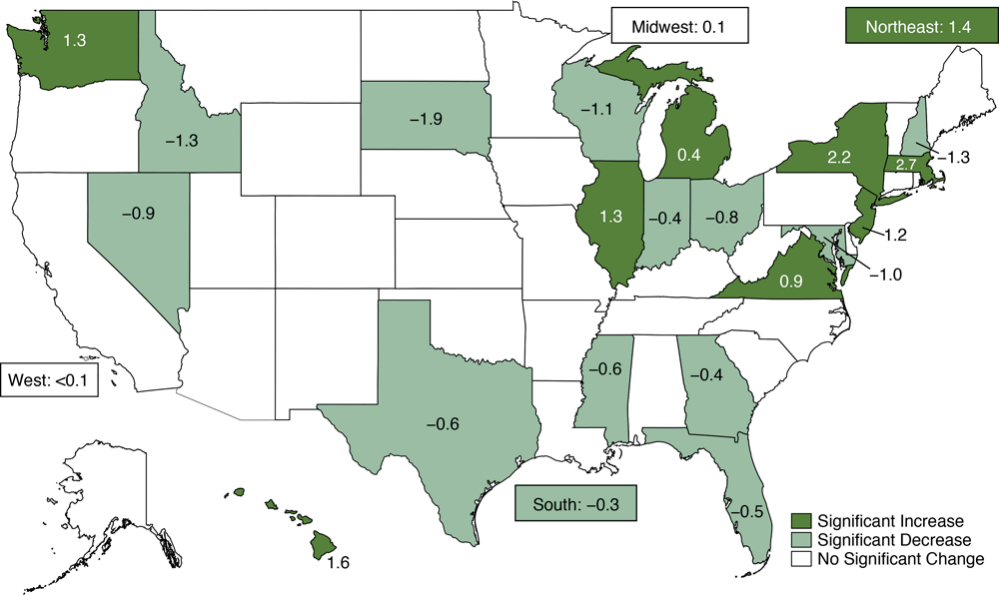
Significant percentage point changes in combined active commuting to work (walking, bicycling, or taking transit) among employed residents aged 16 years or older by state and US Census region, American Community Survey, 2006 to 2017.

**Figure 2:**
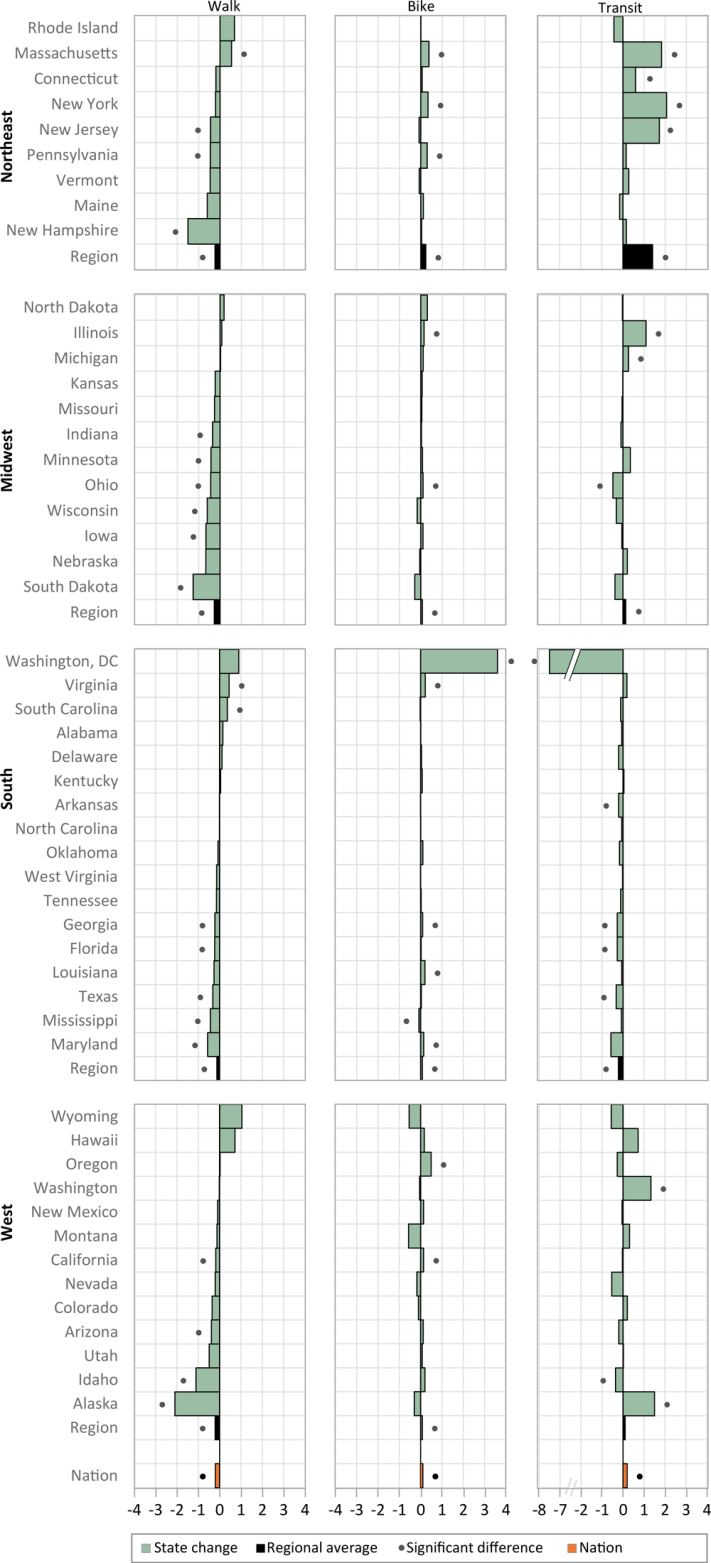
Percentage point changes in walking, bicycling, and taking transit to work among employed residents aged 16 years or older by state and US Census region, American Community Survey, 2006 to 2017.

In the Northeast, the prevalence of active commuting to work increased from 2006 to 2017 in Massachusetts, New York, and New Jersey (Figure 1), and these changes were driven primarily by increases in transit use (Figure 2). Active commuting decreased in New Hampshire, driven by a decrease in walking. Active commuting increased in the Northeast region as a whole, driven by an increase in transit use and offsetting changes in walking and biking.

**Table:** 

Region/State	Walk	Bike	Transit
**North**	**−0.23^a^**	**0.23^a^**	**1.40^a^**
Rhode Island	0.67	0.00	**−**0.43
Massachusetts	0.53^a^	0.38^a^	1.83^a^
Connecticut	**−**0.19	0.07	0.60^a^
New York	**−**0.21	0.34^a^	2.06^a^
New Jersey	**−**0.44^a^	**−**0.08	1.72^a^
Pennsylvania	**−**0.45^a^	0.30^a^	0.15
Vermont	**−**0.46	**−**0.07	0.28
Maine	**−**0.60	0.12	**−**0.15
New Hampshire	**−**1.50^a^	0.04	0.16
**Midwest**	**−0.27^a^**	**0.07^a^**	**0.13^a^**
North Dakota	0.19	0.31	**−**0.03
Illinois	0.09	0.15^a^	1.09^a^
Michigan	0.02	0.11	0.26^a^
Kansas	**−**0.23	0.07	**−**0.01
Missouri	**−**0.26	0.04	**−**0.04
Indiana	**−**0.34^a^	0.03	**−**0.09
Minnesota	**−**0.42^a^	0.08	0.35
Ohio	**−**0.44^a^	0.11^a^	**−**0.48^a^
Wisconsin	**−**0.60^a^	**−**0.17	**−**0.31
Iowa	**−**0.66^a^	0.11	**−**0.05
Nebraska	**−**0.67	**−**0.05	0.21
South Dakota	**−**1.26^a^	**−**0.28	**−**0.38
**South**	**−0.13^a^**	**0.08^a^**	**−0.22^a^**
Washington, District of Columbia	0.90	3.60^a^	**−**3.50^a^
Virginia	0.44^a^	0.22^a^	0.19
South Carolina	0.36^a^	**−**0.02	**−**0.11
Alabama	0.15	0.00	**−**0.06
Delaware	0.11	0.04	**−**0.21
Kentucky	0.04	0.07	0.04
Arkansas	0.00	**−**0.01	**−**0.21^a^
North Carolina	**−**0.01	0.00	**−**0.06
Oklahoma	**−**0.08	0.10	**−**0.17
West Virginia	**−**0.14	0.00	**−**0.03
Tennessee	**−**0.15	0.02	**−**0.11
Georgia	**−**0.22^a^	0.10^a^	**−**0.27^a^
Florida	**−**0.23^a^	0.02	**−**0.27^a^
Louisiana	**−**0.27	0.20^a^	**−**0.07
Texas	**−**0.32^a^	0.03	**−**0.32^a^
Mississippi	**−**0.43^a^	**−**0.09^a^	**−**0.08
Maryland	**−**0.56^a^	0.14^a^	**−**0.58
**West**	**−0.21^a^**	**0.08^a^**	**0.10^a^**
Wyoming	1.04	**−**0.54	**−**0.56
Hawaii	0.72	0.17	0.72
Oregon	0.02	0.49^a^	**−**0.28
Washington	**−**0.02	**−**0.05	1.33^a^
New Mexico	**−**0.09	0.13	**−**0.05
Montana	**−**0.13	**−**0.57	0.31
California	**−**0.18^a^	0.13^a^	**−**0.03
Nevada	**−**0.21	**−**0.18	**−**0.54
Colorado	**−**0.35	**−**0.10	0.20
Arizona	**−**0.39^a^	0.12	**−**0.20
Utah	**−**0.49	0.07	0.02
Idaho	**−**.10^a^	0.20	**−**0.36^a^
Alaska	**−**2.09^a^	**−**0.30	1.50^a^
**Nation**	**−0.20^a^**	**0.10^a^**	**0.20^a^**

^a^ *P* < .05.

In the Midwest, the prevalence of active commuting to work increased from 2006 to 2017 in Illinois and Michigan (Figure 1), both of which were driven primarily by increased transit use (Figure 2). Active commuting decreased in Indiana, Ohio, Wisconsin, and South Dakota. In Indiana, Wisconsin, and South Dakota, decreased active commuting was driven primarily by a decrease in walking. In Ohio, the decrease in active commuting was attributable to decreased walking and transit use, despite an increase in bicycling. In the Midwest region, significant increases in biking and transit were attenuated by decreased walking, resulting in no significant change in combined active commuting.

In the South, active commuting to work increased from 2006 to 2017 in Virginia (Figure 1), where both walking and biking increased (Figure 2). Active commuting decreased in Georgia, Florida, Mississippi, Texas, and Maryland (Figure 1), all of which experienced significant decreases in 2 or 3 of the separate active modes (Figure 2). The observed decrease in combined active commuting in Washington, DC, was large but did not reach significance; a significant increase in biking was attenuated by a significant decrease in transit use (Figure 2). Active commuting decreased in the South, driven by decreases in walking and transit use and despite an increase in biking.

In the West, active commuting to work increased from 2006 to 2017 in Hawaii and Washington (Figure 1); Hawaii experienced nonsignificant increases in all 3 active modes, and transit use increased in Washington (Figure 2). Active commuting decreased in Nevada and Idaho. Nevada had nonsignificant decreases in all 3 active modes, and changes in Idaho were driven primarily by decreased walking. In the West, increased biking was attenuated by decreased walking, resulting in no significant change in combined active commuting.

## Discussion

The prevalence of active commuting to work varied widely among states, from 1.7% in Alabama and Mississippi to 35.0% in New York in 2017. State-level changes from 2006 to 2017 also varied; 8 states exhibited significant increases, and 12 exhibited significant decreases. Changes in the separate active modes also varied by state and contributed to the variation in total active commuting to work. Although these changes were significant, most were of small magnitude; for example, all 3 states with a significant increase in walking to work changed less than 1 percentage point. These results demonstrate how ACS data can be used to monitor and evaluate state-level changes in active commuting.

This analysis extends previous reports of state-level active commuting to work using ACS data (13,14,18) by including all 3 active modes (in aggregate and separately) and including statistical testing of changes over time. For example, previous reports have shown considerable cross-sectional differences in the prevalence of walking and bicycling to work across states, with particularly low prevalence in states from the South Census region (13,18). Our analysis builds on these findings and demonstrates the 4 states in the South with the lowest point estimates for combined active commuting in 2006 experienced either no significant change (Alabama, Arkansas, and Tennessee) or a significant decrease (Mississippi) in combined active commuting. Further, our results suggest the decrease in active commuting in Mississippi was attributed to decreases in both walking and bicycling to work, with no significant change in transit use. These results demonstrate the usefulness of ACS data in highlighting areas for potential improvement.

These results also suggest that including the combined active commuting construct in addition to the 3 separate active modes is valuable when monitoring active commuting to work by using ACS. The combined walking, biking, and transit construct may reveal significant changes in overall active commuting even when there are no significant changes in any of the 3 separate modes that comprise the combined measure. For example, combined active commuting increased significantly in Hawaii, despite having nonsignificant increases in the 3 separate modes. Alternatively, if used in isolation, the combined active commuting construct could obscure 1 or more significant changes in the separate measures of walking, bicycling, and transit use that are in opposite directions (and could therefore “cancel out” when combined). For example, there was no significant change in combined active commuting in Iowa, but the prevalence of walking to work decreased significantly. These results suggest future monitoring efforts might be most valuable when they include combined active commuting to work together with the 3 separate modes.

Although we focused on absolute changes in active commuting to work (ie, prevalence[2017] − prevalence[2006]), other users may benefit from estimating relative changes (ie, [prevalence[2017] − prevalence[2006]] ÷ prevalence[2006]). Relative changes may be particularly important when comparing changes in modes with markedly different prevalence estimates. For example, in New York, bicycling to work increased 0.3 percentage points from 0.4% to 0.7%, which was smaller than the absolute change in taking transit to work (2.0 percentage points: 26.2% [2006] to 28.2% [2017]). However, because the prevalence of bicycling to work was so much lower than that of transit, the relative change in bicycling was larger than the relative change in transit (75% and 8% relative changes, respectively). When comparing changes across modes, relative changes may be an important addition to future monitoring.

Walking to work decreased from 2006 to 2017 in 18 states and nationally, and decreased walking to work contributed to significant reductions in combined active commuting to work in 11 of the 18 states. The reasons for the decreases in walking are not clear. One possibility is commuters shifting between active modes; in 7 states with a significant decrease in walking, there was a concomitant increase in either bicycling or transit use (Alaska, California, Georgia, Maryland, New Jersey, Ohio, and Pennsylvania). Additional research using longitudinal designs, rather than serial cross-sectional analyses, may better explore this possibility.

We chose not to include ACS data from 2005 because they did not include group quarters (eg, college dormitories, military barracks) (19). In post hoc analyses, the prevalence of walking to work among those living in group quarters in 2006 was 35.5%, so 2005 estimates of walking would be lower than that for subsequent years that included group quarters. If 2005 were used as a baseline year, it would inflate change estimates for walking to work. The choice of baseline year is therefore important when monitoring changes in active commuting to work, in particular walking to work, using ACS.

This report focused on state-level monitoring of active commuting, because state-level actions can impact policies, programs, and practices that influence active commuting. For example, the National Complete Streets Coalition of Smart Growth America reports 35 state governments have adopted a Complete Streets policy (6), which helps ensure safe streets for all ages and abilities and balances the needs of different modes (20). Additionally, CDC’s Division of Nutrition, Physical Activity, and Obesity funds state physical activity and nutrition programs across the nation (21) to develop activity-friendly routes to everyday destinations, which is a recommended strategy to increase physical activity, including active transportation (22). Finally, some states have enacted policies or programs to encourage transit-oriented development, which supports active commuting by situating residences, workplaces, and other everyday destinations near public transit stops or stations (10,11). This variety of state-level initiatives that can influence active transportation demonstrates the importance of monitoring changes in state-level estimates of active transportation, including active commuting to work, as one way to evaluate progress toward physical activity goals. State-level public health and transportation professionals can consider using ACS data for this important task.

Although the need for state-level monitoring is clear, state and local professionals may also benefit from estimates at smaller geographic scales. Additional insights about where changes are occurring or evaluation data on local-level policies might be gleaned from estimates at smaller geographic scales, such as counties, incorporated places, or Census tracts. These estimates are available from ACS, but may have large margins of error for rare modes like walking and bicycling, and require combining up to 5 years of ACS data to produce statistically reliable estimates (19). When combining 5 years of data, users would be limited to comparing the average values from 2006–2010 to those of 2013–2017, resulting in only 2 nonoverlapping years in which to detect change. Similarly, estimates at larger geographic scales could be important for providing context to state-specific evaluation of active commuting (eg, Census region–level estimates provided here). For example, professionals in Michigan might interpret a 0.4 percentage point increase in combined active commuting differently when compared with no significant change overall for the Midwest region.

This report is subject to at least 5 limitations. First, all data are self-reported and subject to recall and social desirability biases (23). Second, participants reported only the primary commute mode to work, so those with mixed-mode commutes that include walking, bicycling, or transit as a minor component were not captured. Third, participants only reported the mode of transportation to work, so those who use active modes only for nonwork trips were not counted. Fourth, information on the volume of activity accrued during commutes was not available. Finally, to maximize the time between assessments, we chose to test changes between 2006 and 2017. Future state-level analyses that include intervening years could reveal intermediate changes or shorter-term trends.

This report also has several strengths. First, ACS has a large sample size and a high response rate, which help ensure representativeness. Second, state-level analyses cover populations of sufficient size to use 1-year estimates from ACS, which maximizes the interval over which change can occur. Finally, 1-year estimates allow presentation of the most current estimates of active commuting, without relying on recent multiyear averages.

Active commuting to work remains rare in most states, and changes in active commuting (combined active commuting and the separate modes) have been inconsistent across states; even significant changes have been of modest magnitude. These analyses demonstrate the usefulness of ACS data for state-level monitoring of active commuting to work via walking, bicycling, and using transit. Public health, transportation, and other professionals interested in continued monitoring and evaluation of state-level active commuting to work may find ACS data suitable for this task, and careful consideration of included constructs, change measures, time period, and geographic levels is needed.
